# Use of copeptin in interpretation of the water deprivation test

**DOI:** 10.1002/edm2.399

**Published:** 2023-03-31

**Authors:** Matthew Rowe, Nishchil Patel, Jinny Jeffery, Daniel Flanagan

**Affiliations:** ^1^ Department of Endocrinology University Hospital Plymouth Plymouth UK; ^2^ Combined Labs University Hospitals Plymouth Plymouth UK

**Keywords:** copeptin human, diabetes insipidus, water deprivation

## Abstract

**Introduction:**

Currently, the water deprivation test remains the standard method for distinguishing primary polydipsia (PP) from cranial diabetes insipidus (cDI) and nephrogenic diabetes insipidus (nDI). There is increasing interest in a direct estimate of antidiuretic hormone using plasma copeptin as a stable and reliable surrogate marker. We present our experience of measuring copeptin during the water deprivation test.

**Methods:**

Forty‐seven people (17 men) underwent a standard water deprivation test between 2013 and 2021. Plasma copeptin was measured at the start of the test and at the end of the period of water deprivation (maximum osmotic stimulation). Results were classified using prespecified diagnostic criteria. As it is known that a significant proportion of tests will reveal indeterminate results, a final diagnosis was obtained by including relevant pre‐ and post‐test clinical criteria. This diagnosis was then used to plan individual treatment.

**Results:**

Basal and stimulated copeptin were significantly higher in the nephrogenic DI group than other categories (*p* < .001). There was no significant difference in basal or stimulated copeptin between PP, cDI or partial DI. Nine results were indeterminate where the serum and urine osmolality did not give a unified diagnosis. Stimulated copeptin was helpful in reclassifying these patients into the final diagnostic groups.

**Conclusion:**

Plasma copeptin has additional clinical utility in interpretation of the water deprivation test and may continue to have a place alongside newer stimulation tests.

## Graphical abstract

The water deprivation test has, for many years, been the standard approach to investigating the polyuria/polydipsia syndrome. Our results suggest there is additional clinical value in adding the measurement of copeptin to this test.

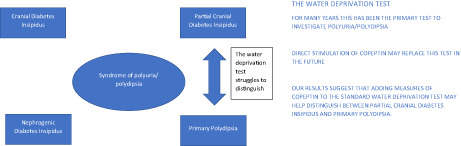

## 1 INTRODUCTION

For many years, the water deprivation test has been the standard method for differentiating between the causes of the polyuria/ polydipsia syndrome.^1^ The test will distinguish between diabetes insipidus and the syndrome of primary polydipsia (PP). With cranial diabetes insipidus (cDI), there is a deficiency of antidiuretic hormone (ADH), also known as arginine vasopressin (AVP) following damage to the hypothalamus or posterior pituitary, resulting in an inability to retain water and concentrate urine. With PP, there is an urge to drink excessively that results in high urine output. It is recognized that there may be partial deficiency of ADH—partial cranial diabetes insipidus (pDI) resulting in abnormal but not total inability to concentrate urine. There are a smaller group of people who develop resistance to the action of antidiuretic hormone at the level of the kidney (nephrogenic DI). It is important to distinguish between PP and cDI as the management of the two conditions is significantly different.^2^, ^3^ The major difference between the two conditions is the ability to release ADH in response to osmotic stimulation but the assay is time‐consuming.^4^, ^5^ In the absence of a significant increase in serum osmolality, measured concentrations are often very low. The water deprivation test involves a medically supervised period of water restriction where the individual's weight, urine output, serum and urine osmolalities are measured. For a significant proportion of people, the test will be equivocal with some concentration of the urine but not achieving the established cut‐off for normality. This may be because there is partial impairment of ADH production or because large volumes of dilute urine associated with polydipsia will degrade the ability of kidney to produce concentrated urine by reducing the renal medullary concentration gradient.^6^ Although the test is widely used, it has significant drawbacks and is an indirect way of diagnosing ADH deficiency.

In recent years, an immuno‐assay has been developed for the measurement of copeptin, a fragment of the preprovasopressin molecule.^4^, ^7^ Copeptin is produced in equimolar amounts to ADH and provides a more direct method for diagnosing diabetes insipidus.

Although measures of stimulated copeptin may supersede this, the water deprivation test may still have a place. Hypertonic saline will directly stimulate copeptin but may be contraindicated in some groups such as those with cardiac failure, risk of stroke or epilepsy in which case the water deprivation test may still be needed. Arginine infusion may be used as an alternative but also has some contraindications. If the water deprivation test continues to have some use in the future, it is possible that adding a basal and stimulated copeptin might increase the accuracy of the test. We present a retrospective analysis of the utility of adding copeptin to the water deprivation test.

## 2 METHODS

This was a retrospective single centre‐based study involving 47 people who underwent a standardized water deprivation test between 2013 and 2021. All patients had been referred for investigation of polyuria/ polydipsia with the differential diagnosis including diabetes insipidus or primary polydipsia. Prior to the test, other causes such as diabetes mellitus, hypercalcaemia, renal insufficiency or other electrolyte disturbance had been excluded.

The patients were admitted to the endocrine investigation unit at 08.00 on the morning of the test. The protocol is summarized below: fluid was not restricted before arrival in the unit. Consumption of any liquids during the test was prohibited, and patients were not allowed to leave the ward. Body weight, serum and urine osmolality, electrolytes and copeptin were measured at the start of the test. Serum and urine osmolality and urine volume were recorded every 90 minutes. Copeptin was repeated at the end of 6 h of water restriction, at which point a decision was made, based on the serum and urine osmolality and urine volumes, whether to proceed with desmopressin. Weight was measured every 2 h. If desmopressin was administered, serum and urine osmolality were followed hourly for the next 4 h. The test was concluded if, at any point, body weight fell by >5% or the urine osmolality exceeded 700 mmol/kg (at which point serum and urine osmolality, weight and electrolytes were measured).

The criteria developed for the interpretation of the water deprivation test are shown in Table 1. The results for each test were reviewed by the multidisciplinary endocrine team. Based on the presenting clinical picture and results of water deprivation, a provisional diagnosis was determined. There were four potential diagnoses: cranial diabetes insipidus (cDI), partial diabetes insipidus (pDI), primary polydipsia (PP), which is essentially an ability to normally concentrate urine in the face of water restriction, and nephrogenic diabetes insipidus (nephrogenic DI). Previous data have suggested that the diagnosis of diabetes insipidus could be improved by the addition of copeptin measurement.^8^ Suggested copeptin diagnostic cut‐offs derived from previous studies are also included in Table 1. It is accepted that the water deprivation test can produce equivocal results particularly in distinguishing pDI and PP. It is usual practice in these situations to combine the results of the water deprivation test with other clinical information to conclude a presumptive diagnosis. This might then involve a trial of desmopressin or advice about fluid consumption and continued clinical observation. Following a variable period of follow‐up, a substantive diagnosis is then reached. The patient may continue to be followed or discharged depending upon the clinical course. Although urine volumes and weight were recorded as part of the test, these were kept as paper records and were not available for analysis in this cohort.

**TABLE 1:** Diagnostic criteria for the water deprivation test

	Primary polydipsia (normal concentrating ability)	Diabetes Insipidus	Partial Diabetes Insipidus	Nephrogenic Diabetes Insipidus
Peak Serum Osmolality mmol/kg	< 295	>295	>295	>295
Peak Urine Osmolality mmol/kg	>600	<300	300–600	<300
Change in urine osmolality Postdesmopressin	<50% rise	>50% rise	9–50% rise	<10% rise
Additional criteria for Copeptin interpretation	>4.9 pmol/L	<2.6 pmol/L	2.6–4.9	>20 pmol/L

Copeptin was measured using the Brahms CT‐proAVP Kryptor assay. According to the manufacturer's instruction for use, the limit of detection was assessed as being 1.2 pmoL/L, the interassay coefficient of variation as being 5.5% for mean copeptin concentrations of an internal quality control material at a concentration of 5.4 pmoL/L, 5.1% for a serum pool with a copeptin concentrations of 16.9 pmoL/L and 3.5% for a quality control material at a concentration of 102.6 pmoL/L.

Differences between groups were compared using one‐way analysis of variance (SPPS vs 25). The study was approved and registered with the audit department at University Hospitals Plymouth NHS Trust CA_2020–21‐019. Ethical review and consent were not required for this study.

## 3 RESULTS

Between 2013 and 2021, 47 consecutive water deprivation tests were performed at University Hospital Plymouth. Copeptin was measured at baseline and at the end of a 6‐h period of water deprivation for each subject. Details of the clinical cohort are displayed in Table 2.

**TABLE 2:** Clinical characteristics of the 47 patients undergoing the water deprivation test

	Primary Polydipsia	Diabetes Insipidus	Partial Diabetes Insipidus	Nephrogenic Diabetes Insipidus
*N*	24	8	11	4
Gender (female/male)	16/8	4/4	7/4	3/1
Age mean (SD)	52 (14)	57 (17)	55 (19)	64 (11)
Age median (IQR)	51 (22)	63 (30)	62 (28)	62 (21)
Evidence of hypothalamic/ pituitary infundibulum/ pituitary abnormality on imaging	3/12	8/8	9/10	‐
Anterior pituitary hormone dysfunction	4/21	8/8	3/10	‐
Lithium treatment	–	–	–	3
Polycystic kidney disease	–	–	–	1
Long‐term desmopressin treatment	0/26	9/9	6/9	0

All the subjects described had presented with a syndrome of polyuria and polydipsia. All had demonstrated high volumes of dilute urine production. The differential diagnosis for this presentation includes cDI, pDI, PP or nephrogenic DI. It is accepted that the water deprivation test alone can struggle to accurately define a specific diagnosis in all cases. There is often a discrepancy in the diagnostic criteria with, for example, an individual maintaining a normal serum osmolality with water deprivation but not achieving the urine concentration required to make a diagnosis of PP or alternatively showing an ability to adequately concentrate the urine but also showing a serum osmolality that climbs above the normal range. The water deprivation test therefore requires interpretation in the clinical context for that individual considering their medical history and subsequent clinical course. The four diagnostic groups shown in Table 2 are based on the tests results together with 1–8 years of clinical follow‐up. The largest group were those with a final diagnosis of PP (*n* = 24), and pDI was the next largest group (*n* = 11). There were relatively few cases of cDI (*n* = 8) tested this may reflect the fact that in the correct clinical context (for example following pituitary surgery) patients may be directly started on desmopressin therapy based on close observation of post‐operative fluid balance and electrolytes and do not proceed to formal water deprivation. There were no significant age or gender differences between the groups. All the patients with cDI had anatomical abnormality on imaging that could account for the condition. Four of these had conditions where there was the possibility of the pituitary function changing with time (pituitary apoplexy, hypophysitis and lymphoma). Ten of 11 people with pDI had imaging available, nine of these had an imaging abnormality. Three of these individuals had pituitary stalk abnormalities with the possibility of a change in the clinical condition with time. The remaining individual had normal pituitary/ hypothalamic imaging but had had significant head trauma. All the patients with nephrogenic DI were suspected to have the condition prior to the test either due to lithium treatment or due to polycystic kidney disease.

**FIGURE 1:**
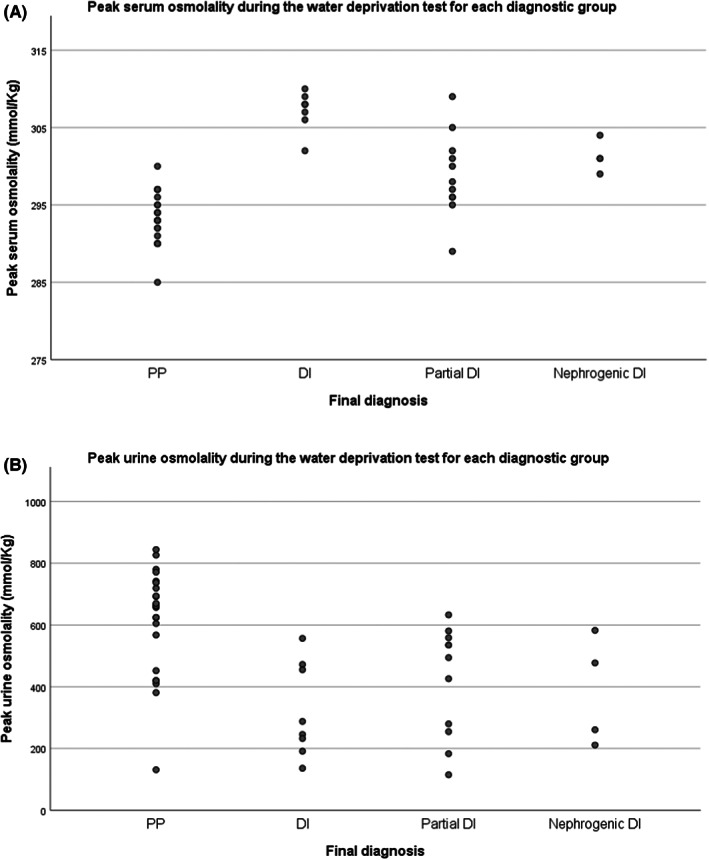
Peak (A) serum, (B) urine osmolality during the water deprivation test for each diagnostic group. Graph of baseline copeptin measurement against the final diagnosis for each patient. P for difference between Nephrogenic DI and others *p* < .001. No other significant differences between groups.

Figures 1A,B show the peak serum and urine osmolality measured during the 6 h of water deprivation for each individual. Results are grouped by the final diagnosis based on both the overall water deprivation tests results, the background condition and subsequent course. Summary statistics show a significant difference between groups for both serum and urine results (*p* < .001). There were significant differences between pDI and PP for peak serum osmolality (*p* = .007) and for peak urine osmolality (*p* = .015). Group statistics are unhelpful when determining the usefulness of the test for an individual patient. Seven people with a find diagnosis of PP did not achieve a peak urine osmolality of >600 mmol/kg although serum osmolality remained normal. Four individuals with PP achieved a peak serum osmolality of >295 mmol/kg all these people achieved a urine concentration > 600 mmol/kg (consistent with PP). In the group with final diagnosis of pDI, one person did not achieve a serum osmolality >295 although their urine remained dilute (peak urine osmolality 115 mmol/kg). One individual in the pDI group achieved a peak urine osmolality of 633 mmol/kg, outside of the diagnostic criteria, but this was at a time when serum osmolality had reached 302 mmol/kg.

Eighteen individuals proceeded to desmopressin administration following the period of water deprivation. None of the pp group achieved a 50% rise in urine osmolality. All of cDI group except one achieved a 50% rise, in that individual the serum osmolality had climbed to 305 with water restriction and normalized with desmopressin and drinking. The pDI group showed a variable response to desmopressin with a range of 18–200% increase.

**FIGURE 2:**
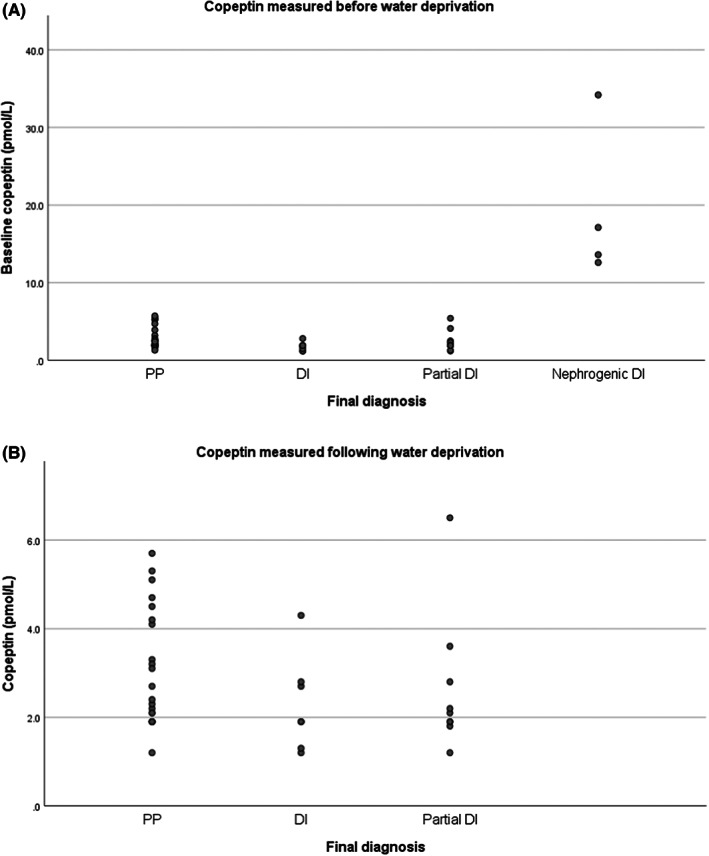
Copeptin measured (A) before, (B) following water deprivation.

**FIGURE 3:**
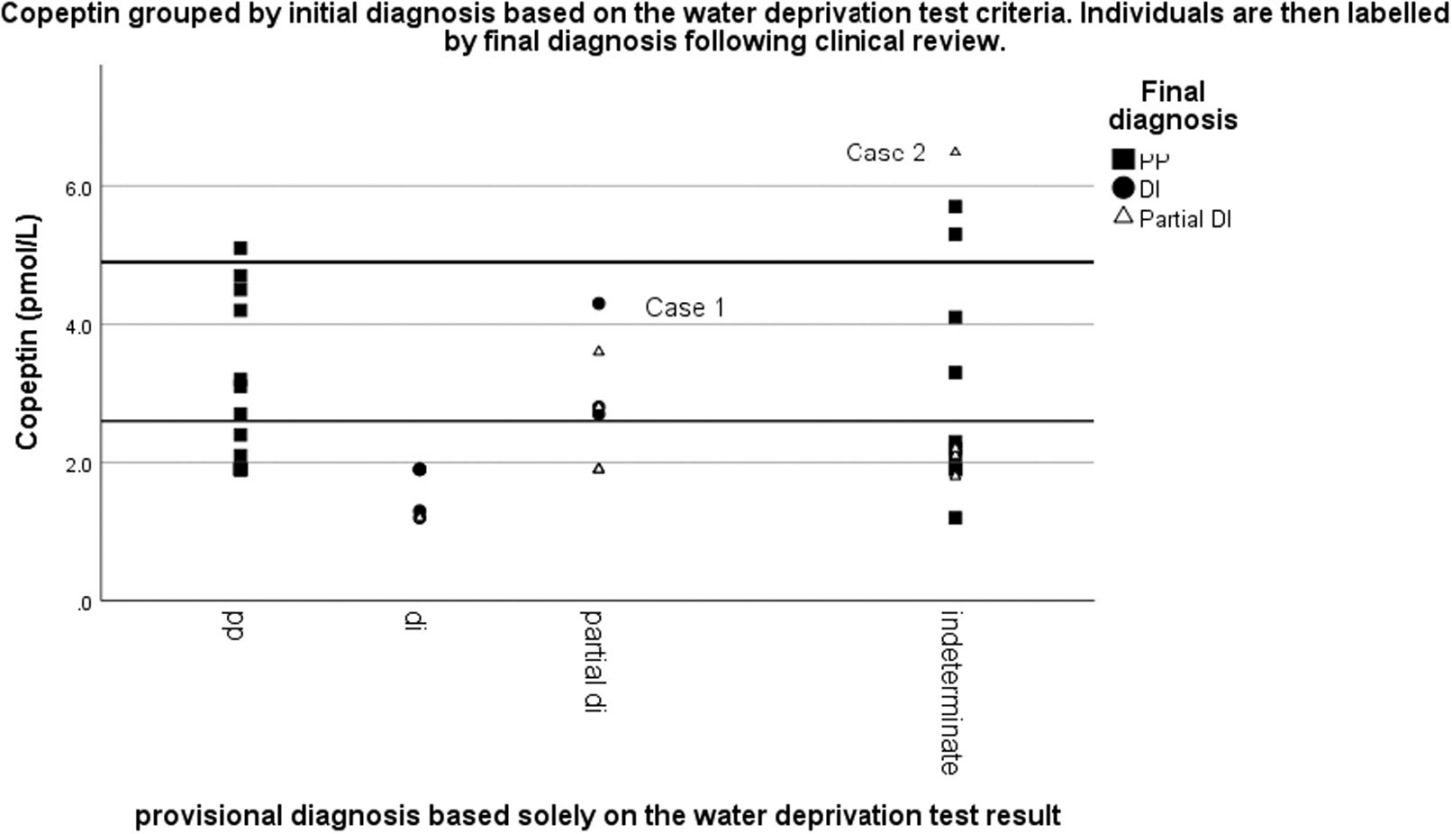
Copeptin grouped by initial diagnosis based on the water deprivation test criteira. Individuals are then labelled by final diagnosis following clinical review. Graph showing the utility of measuring stimulated copeptin for those patients with indeterminate results following the water deprivation test. Results are grouped by the final diagnosis taking into account the full clinical circumstances of each case.

Figure 2 shows the individual values of copeptin measured before the period of water deprivation. There is a clinically and statistically significant difference between copeptin values for nephrogenic DI and the other groups (*p* < .001); otherwise, there were no significant differences between groups. Figure 2B shows the individual copeptin values following a period of water deprivation. As the values of copeptin are so much higher for the nephrogenic DI group, they have been omitted from the figure to emphasize the differences between the other three diagnoses. Again, there is no significant difference between groups. The groups that do get more dehydrated (cDI and pDI) are those with defects in copeptin release and also do not show a significant rise. There are two individuals (one in the cDI group and one in the pDI group) who show an unexpected rise in copeptin despite having a confirmed diagnosis of presumptive antidiuretic hormone deficiency. These two cases are discussed further below. Figure 3 displays the stimulated copeptin measurements by the diagnosis for individuals based solely on the water deprivation test. Individuals can either meet the required diagnostic criteria based on serum and urine osmolalities to make a specific diagnosis or are otherwise classified as indeterminate if the results are discrepant. Each individual is then labelled by the final diagnosis achieved based on clinical context and further clinical review. Copeptin cut‐offs at 2.6 and 4.8 pmol/L have been added (derived from previous publications).^8^, ^9^ There is a wide spread of copeptin values within the PP group, but each individual had already achieved a firm diagnosis based on serum and urine osmolality changes. Most patients with a final diagnosis of cDI achieved a clear diagnosis during the water deprivation test although two individuals would have been classified as pDI based solely on the test. The addition of copeptin would have helped to clarify the diagnosis for one of these two but the individual labelled as Case 1 achieved a stimulated copeptin outside of the suggested range for a diagnosis of cDI.

Case 1 presented in pregnancy with presumed hypophisitis. Her water deprivation test at that point was diagnostic of cDI, and she was replaced with desmopressin with resolution of her symptoms. Her water deprivation test was repeated 7 years later to look for improvement, on this occasion copeptin was included. This again clearly showed cDI with a peak serum osmolality of 309 mmol/kg and a peak urine osmolality of 472 mmol/kg. The urine concentrated to 701 mmol/kg with desmopressin. This fulfils the criteria for cDI with the implication being that she is deficient in antidiuretic hormone but her copeptin result at 4.3 pmol/L suggested at least some residual antidiuretic hormone production.

Case 2 was an 88‐year‐old man who had presented with pituitary apoplexy and diabetes insipidus. He had been started on anterior pituitary hormone replacement and desmopressin as an inpatient. Following water deprivation, there was no change in the serum osmolality but also no change in the urine osmolality with a peak urine osmolality of 115 mmol/Kg. Following desmopressin, the urine concentrated to 402 mmol/Kg. His stimulated copeptin was comfortably normal at 6.5 pmol/L despite not seeing a significant change in his serum concentration. He was discharged on a continuing dose of desmopressin and unfortunately readmitted to hospital 3 months later with a dilutional hyponatraemia that responded to a reduction in desmopressin dose. He had responded to desmopressin treatment, but had normal copeptin suggesting relative deficiency together with some resistance to hormone action.

## 4 CONCLUSION

We have studied the utility of adding copeptin measurement at baseline and following water deprivation as an addition. Nephrogenic diabetes has significantly higher copeptin measurements on both baseline and stimulated samples. This diagnosis can be confirmed on a baseline blood test, and water deprivation may not be required. To distinguish between cDI, pDI and PP, the water deprivation test showed significant differences in both serum and urine osmolality between groups and specifically between pDI and PP. Stimulated or baseline copeptin, however, did not show a significant difference between groups (excluding nephrogenic DI). This does not imply that adding copeptin does not have some clinical utility when added to the water deprivation test. It has previously been established that a significant proportion of test results will be indeterminate^8^ and this was true for this cohort. For the indeterminate group in this cohort, the final clinical diagnosis for the majority was primary polydipsia with a smaller number being diagnosed with partial DI. The copeptin measurements for all but one person were below the suggested cut‐off for diagnosing pDI. The copeptin measurement was therefore helpful in these cases in confirming the final diagnosis. There was one patient (Case 2) in the indeterminate group where the diagnosis of pDI was strongly suspected based on the clinical picture and measured osmolalities (and had resolved with desmopressin therapy), but the copeptin measure was unexpectedly high. In this case, the significance of the unusually high copeptin is not clear. In retrospect close follow‐up of this unusual result might have prevented his subsequent development of dilutional hyponatraemia. A second case with a diagnosis of partial DI based solely on the water deprivation test had a final diagnosis of cDI based on the clinical picture and remained on life‐long desmopressin therapy despite a copeptin measurement significantly above the suggested cut‐off for this condition. Copeptin stimulation tests using hypertonic saline or arginine may have misdiagnosed these cases and emphasizes the importance of interpreting the results in the clinical context.

Different units use variations of the test with variations in the period of water deprivation. The period of water deprivation used in our unit is shorter than in some previous publications. The exact length of fluid deprivation is a balance between ensuring an adequate stress while avoiding unnecessary risk to the individual. There is also the practical consideration of performing the test within a single day and avoiding the need for admission to a hospital bed. Fenske and colleagues^9^ published the results of copeptin measurement in a similar cohort measuring baseline and stimulated copeptin following water deprivation. There were some differences in the water deprivation protocol (with longer fluid restriction for a proportion). There were also some differences in the osmolality cut‐offs defining the diagnoses. The conclusions of the study were broadly similar; adding copeptin produces a modest improvement in diagnostic accuracy. Their cohort did not produce any examples of unexpectedly high copeptin measurement in individuals with a diagnosis of diabetes insipidus or partial diabetes insipidus. Subsequent studies have shown that the test could be improved by the addition of water deprivation with hypertonic saline.^10^ Since then, this has developed into hypertonic saline stimulation alone or, more recently, arginine stimulation.^8^, ^11^, ^12^ Both new tests look very promising with significantly improved diagnostic accuracy although the tests may not be suitable for all patients.

One of the strengths of these data is that they represent real‐world data with a relatively long period of follow‐up for most subjects after the test. The test of time helps in ensuring that the final diagnosis is secure.

A limitation of this study is the lack of information about urine volumes or change in weight over the length of the test. This information would have been available to the clinicians when interpreting the results and have been factored into the presumptive diagnosis but are not available to include in this paper. A further difficulty with using any test to diagnose ADH deficiency is that it appears to be a hormone axis where we see more variation in function with time. It is common to see transient diabetes insipidus after surgery that recovers in a few days.^13^, ^14^ Similarly with pituitary haemorrhage or infarction, DI may be transient over a longer period.^15^ With inflammatory pituitary or hypothalamic lesions, DI may fluctuate with periods of recovery and then recurrence.^16^ This can make the final diagnosis more elusive as it possible that it may change after the diagnostic test has been performed.

Our data would also perhaps suggest that there will be individuals where the copeptin appears normal on stimulation, but the person demonstrates an inability to concentrate urine that is reversed with desmopressin therapy. It is important to be aware of this if clinical practice switches primarily to direct stimulation test. The future paradigm may require a water deprivation test with copeptin measurement for these individuals where there is a very strong clinical suspicion despite normal copeptin stimulation.

## AUTHOR CONTRIBUTIONS

**Matthew Rowe:** Data curation (equal); investigation (equal); writing – review and editing (equal). **Nishchil Patel:** Data curation (equal); formal analysis (equal); investigation (equal); writing – review and editing (equal).

## CONFLICT OF INTEREST

The authors have no conflicts of interest to declare.

## Data Availability

The data that support the findings of this study are available on request from the corresponding author. The data are not publicly available due to privacy or ethical restrictions.

## References

[1] Miller M , Dalakos T , Moses AM , Fellerman H , Streeten DH . Recognition of partial defects in antidiuretic hormone secretion. Ann Intern Med. 1970;73(5):721‐729. 5476203 10.7326/0003-4819-73-5-721

[2] Fenske W , Allolio B . Clinical review: current state and future perspectives in the diagnosis of diabetes insipidus: a clinical review. J Clin Endocrinol Metab. 2012;97(10):3426‐3437. 22855338 10.1210/jc.2012-1981

[3] Qureshi S , Galiveeti S , Bichet DG , Roth J . Diabetes insipidus: celebrating a century of vasopressin therapy. Endocrinology. 2014;155(12):4605‐4621. 25211589 10.1210/en.2014-1385

[4] Morgenthaler NG , Struck J , Alonso C , Bergmann A . Assay for the measurement of copeptin, a stable peptide derived from the precursor of vasopressin. Clin Chem. 2006;52(1):112‐119. 16269513 10.1373/clinchem.2005.060038

[5] Robertson GL , Mahr EA , Athar S , Sinha T . Development and clinical application of a new method for the radioimmunoassay of arginine vasopressin in human plasma. J Clin Invest. 1973;52(9):2340‐2352. 4727463 10.1172/JCI107423 PMC333039

[6] Li C , Wang W , Kwon TH , et al. Downregulation of AQP1, −2, and −3 after ureteral obstruction is associated with a long‐term urine‐concentrating defect. Am J Physiol Renal Physiol. 2001;281(1):F163‐F171. 11399657 10.1152/ajprenal.2001.281.1.F163

[7] Balanescu S , Kopp P , Gaskill MB , Morgenthaler NG , Schindler C , Rutishauser J . Correlation of plasma copeptin and vasopressin concentrations in hypo‐, iso‐, and hyperosmolar states. J Clin Endocrinol Metab. 2011;96(4):1046‐1052. 21289257 10.1210/jc.2010-2499

[8] Fenske W , Refardt J , Chifu I , et al. A Copeptin‐based approach in the diagnosis of diabetes insipidus. N Engl J Med. 2018;379(5):428‐439. 30067922 10.1056/NEJMoa1803760

[9] Fenske W , Quinkler M , Lorenz D , et al. Copeptin in the differential diagnosis of the polydipsia‐polyuria syndrome–revisiting the direct and indirect water deprivation tests. J Clin Endocrinol Metab. 2011;96(5):1506‐1515. 21367924 10.1210/jc.2010-2345

[10] Timper K , Fenske W , Kühn F , et al. Diagnostic accuracy of Copeptin in the differential diagnosis of the polyuria‐polydipsia syndrome: a prospective multicenter study. J Clin Endocrinol Metab. 2015;100(6):2268‐2274. 25768671 10.1210/jc.2014-4507

[11] Bologna K , Cesana‐Nigro N , Refardt J , et al. Effect of arginine on the hypothalamic‐pituitary‐adrenal Axis in individuals with and without vasopressin deficiency. J Clin Endocrinol Metab. 2020;105(7):e2327‐e2336. 10.1210/clinem/dgaa157 32236441

[12] Winzeler B , Cesana‐Nigro N , Refardt J , et al. Arginine‐stimulated copeptin measurements in the differential diagnosis of diabetes insipidus: a prospective diagnostic study. Lancet. 2019;394(10198):587‐595. 31303316 10.1016/S0140-6736(19)31255-3

[13] Joshi RS , Pereira MP , Osorio RC , et al. Identifying risk factors for postoperative diabetes insipidus in more than 2500 patients undergoing transsphenoidal surgery: a single‐institution experience. J Neurosurg. 2022;137:1‐11. 10.3171/2021.11.JNS211260 35090129

[14] Kim YH , Kim YH , Je YS , Lee KR , Lim HS , Kim JH . Changes in copeptin levels before and 3 months after transsphenoidal surgery according to the presence of postoperative central diabetes insipidus. Sci Rep. 2021;11(1):17240. 34446748 10.1038/s41598-021-95500-x PMC8390481

[15] Iqbal F , Adams W , Dimitropoulos I , Muquit S , Flanagan D . Pituitary haemorrhage and infarction: the spectrum of disease. Endocr Connect. 2021;10(2):171‐179. 33434143 10.1530/EC-20-0545 PMC7983520

[16] Català Bauset M , Gilsanz Peral A , Girbés Borràs J , et al. Clinical practice guideline for the diagnosis and treatment of hypophysitis. Endocrinol Nutr. 2008;55(1):44‐53. 22967850 10.1016/S1575-0922(08)70634-X

